# Ultrasonic deterrents provide no additional benefit over curtailment in reducing bat fatalities at an Ohio wind energy facility

**DOI:** 10.1371/journal.pone.0318451

**Published:** 2025-05-08

**Authors:** Jeff Clerc, Manuela Huso, Michael Schirmacher, Michael Whitby, Cris Hein

**Affiliations:** 1 National Renewable Energy Laboratory, Golden, Colorado, United States of America; 2 United States Geological Survey, Forest and Rangeland Ecosystem Science Center, Corvallis, Oregon, United States of America; 3 Copperhead Environmental Consulting, Inc., Paint Lick, Kentucky, United States of America; 4 Bat Conservation International, Austin, Texas, United States of America; King Fahd University of Petroleum & Minerals, SAUDI ARABIA

## Abstract

Wind energy is important for achieving net-zero greenhouse gas emissions but also contributes to global bat mortality. Current strategies to minimize bat mortality due to collision with wind-turbine blades fall broadly into two categories: curtailment (limiting turbine operation during high-risk periods) and deterrence (discouraging bat activity near turbines). Recently, there has been interest in combining these strategies to achieve greater reductions in bat fatalities than either strategy might achieve in isolation. To investigate the effectiveness of combining curtailment with ultrasonic deterrent minimization strategies, we deployed six ultrasonic deterrents at nacelle height on 16 experimental turbines at Avangrid Renewables’ Blue Creek Wind Energy Facility. We rotated between four conditions (normal operations, curtailment only, deterrent only, curtailment and deterrent) randomly assigned to four wind turbines each night between 15 June and 3 October 2017. We found that bat mortality at wind turbines was independent of wind speed. The effectiveness of ultrasonic acoustic deterrents varied between high-frequency-calling species (eastern red bats) and low-frequency-calling species (hoary bats, silver-haired bats, and big brown bats). When deterrents were active, mortality was twice as high for eastern red bats compared to the control. Conversely, deterrents had a weak dampening effect on bat mortality for low-frequency species. We found no additive effects on mortality reduction for turbines operating both curtailment and deterrents compared to either approach in isolation. Our findings suggest that ultrasonic acoustic deterrents may not be effective for both high and low frequency echolocating bats. The increase in fatalities of eastern red bats is alarming and underscores the importance of considering site- and species-specific effects of minimization solutions.

## Background

To achieve net-zero greenhouse gas emissions, wind energy is projected to expand from 5% of global power generation to 35%, and greater than 50% of future electricity demand [1]. Despite wind energy being a foundation energy source of future integrated energy systems [2], wind turbines remain a major contributor to annual global bat mortality [3–5]. In the United States and Canada, hundreds of thousands of bat fatalities are estimated to occur at wind energy facilities annually [3]. For species particularly susceptible to wind turbine collisions, such as hoary bats (Lasiurus cinereus), fatalities related to wind energy have the potential to affect populations if minimization approaches are not widely adopted by wind energy facility operators [5–6].

Our current understanding of the mechanisms by which bats may be attracted to and subsequently killed by wind turbines is incomplete [7–9]. However, our growing knowledge of the conditions associated with increased bat mortality (e.g., seasonality, wind speeds) has led to investigations of several minimization approaches that focus on curtailing wind turbines during specific times and weather conditions or deterring bats from approaching wind turbines. Curtailment involves rotating blades parallel to the prevailing wind, such that the blades rotate less than one full rotation per minute when the risk of bat mortality is high. The most common curtailment approach, often called blanket or wind-only curtailment, prevents turbine rotation when winds are below a designated curtailment speed to minimize exposure (i.e., the temporal and spatial overlap between bat activity and operating turbines). Alternatively, deterrence aims to limit exposure by suppressing bat activity near operating wind turbines, regardless of site conditions. Several deterrents have been investigated, including ultrasonic [3,10], radar [11], and dim ultraviolet luminescence [12,13]. Ultrasonic deterrents that emit high-frequency noise have been the most widely tested technique at wind energy facilities.

Wind-only curtailment has been effective in reducing bat mortality by an average of 62% (95% confidence interval (CI): 54%–69%), when turbines are curtailed below 5.0 m/s [14]. Restricting curtailment to conditions when collision risk is high will help minimize energy generation losses. Alternatives to wind-only curtailment designed to minimize energy losses incorporate additional biotic covariates (e.g., bat activity rates) and/or abiotic covariates (e.g., temperature) to better predict collision risk [15,16]. Yet the ability to predict risk remains equivocal, and to date, wind-only curtailment remains the most widely accepted and implemented curtailment approach. Since any curtailment results in energy generation loss, there is continued interest in developing deterrent technologies that are as effective or more effective than curtailment. However, results are mixed among studies measuring bat mortality reduction due to ultrasonic deterrents [10,17,18], the drivers of which remain unclear. For example, Weaver et al. [10] found fewer hoary bat (treatment: n = 8, control: n = 37) and Brazilian free-tailed bat (*Tadarida brasiliensis*) (treatment: n = 152, control: n = 334) carcasses at turbines with ultrasonic deterrents relative to control turbines but found no difference in the number of northern yellow bat (*Lasiurus intermedius*) carcasses (treatment: n = 25, control: n = 20).

Our limited understanding of population level effects of wind energy mortality on bats indicates that a greater reduction in fatality may be needed than is widely available today with low wind speed curtailment. In the absence of a perfect solution, the idea of combining curtailment and deterrent strategies has received recent interest (e.g., [18]). By combining minimization strategies, researchers seek to investigate the potential for synergistic effects that provide greater overall fatality reductions than those achieved by a single approach. In 2017, we conducted a study to test potential synergistic effects to minimize bat mortality by combining curtailment and ultrasonic deterrent minimization measures.

The analysis and comparison of curtailment has challenges related to the wind speed-based implementation. A wind turbine assigned to a curtailment treatment will operate differently from one assigned to be fully operational only at specific wind speeds. At other times, turbine operations are identical. The time curtailment is implemented varies between years at the same site and among sites based on that season’s specific wind regime. Thus, the difference in operation between turbines with and without curtailment and their expected differences in bat mortality will strongly depend on (i) the proportion of the night for which wind speed is below or above the curtailment threshold and (ii) whether or not this part of the night coincides with bat activity.

Therefore, one goal of our study was to determine if there is a relationship between wind speed and bat fatalities. We hypothesized that bat fatalities are directly related to the proportion of the night during which turbines were spinning. When that proportion is close to 0, mortality should be close to 0. As the proportion of time the wind turbines are spinning approaches 1, mortality should be at its maximum. However, there could be considerable variability in mortality at this upper end, depending on actual wind speed, its influence on bat activity, and the randomness of encounters with turbines.

Our study’s primary goal was to determine the degree of fatality reduction relative to control that was associated with three minimization treatments: Curtailment to 5.0 m/s, Ultrasonic Deterrents, and the combination of the two. We hypothesized:

- Fatality reduction resulting from the curtailment treatment will be in proportion to the amount of time during which turbines were spinning during the night.
- Ultrasonic deterrents will reduce fatality levels, but results may be species-specific.
- Combining ultrasonic deterrents and curtailment will reduce bat fatalities more than either treatment alone.

## Methods

### Study site

We conducted this study at Avangrid Renewables’ Blue Creek Wind Energy Facility (hereafter, Blue Creek) in Van Wert and Paulding counties, Ohio. Blue Creek is a 304-MW facility located in an agricultural setting and has been operational since October 2011. The site consists of 152 2.0-MW Gamesa G90 wind turbines, each with a 100-m hub height and 90-m rotor diameter. The turbines are designed to begin producing energy (cut in) when winds are above 3.0 m/s with rotation speed of 9 revolutions per minute (RPM), achieve maximum rotation speed (19 RPM) when winds are between 6.0 and 8.0 m/s, and continue rotating at 19 RPM until winds are ≥ 21.0 m/s, at which point turbines are cut out to prevent damage to the turbines. Tip speed at cut-in (9 RPM) is 153 km/h, and maximum tip speed (at 19 RPM) is 322 km/h). At this site, all turbines were feathered (<2 RPM; maximum tip speed 34 km/h) when winds were below the manufacturer’s cut-in speed of 3 m/s.

Blue Creek is an ideal study site for several reasons, including the site’s long history of post-construction monitoring (useful for developing the experimental design), high visibility for searching, ability to clear large search plots, and comparable wind conditions and reported bat fatality rates within the Midwest. Turbines at Blue Creek are relatively evenly distributed within the facility footprint. The 16 experimental turbines were randomly distributed throughout the facility with turbines having a minimum distance of 250 m.

### Treatments

#### Ultrasonic deterrent deployment.

Ultrasonic deterrents intend to discourage bats within the ensonified area by creating a sound that interferes with echolocation capabilities. This sound makes it difficult to forage and navigate thus causing bats to avoid the area. We used the NRG bat deterrent (NRG Systems, Hinesburg, VT). This deterrent uses 6 piezoelectric transducers to emit a constant sound ranging from approximately 50–120 kHz. The sound pressure level, measured at 1 m from the transducers is 12 decibels.

We installed six ultrasonic deterrents at nacelle height on all 16 experimental turbines, four on the top of the nacelle and two on the underside of the nacelle, which we could activate or not on a nightly basis. We secured three on the top of the turbine to the fiberglass housing of the nacelle using bracketry while the fourth was bolted to the weather mast. All four topside ultrasonic deterrents used cables that passed through the supplied cable inlets. We mounted two ultrasonic deterrents to the bottom of the nacelle, one in the upwind position near the main bearing and another in the downwind position, both pointing downward.

#### Curtailment.

Rotation of experimental wind turbines was curtailed when the average wind speed measured over a 10-minute period at a single meteorological tower on the site was below 5.0 m/s. In addition, rotation of all wind turbines at the site, including experimental ones, was curtailed when the average wind speed was below the manufacturer’s cut-in speed of 3 m/s. At the end of the study, the operator provided data representing 10-minute averages of meteorological and operational variables recorded at this meteorological tower and at each experimental turbine. These data included average wind speed, temperature, and precipitation measured at the met tower, and average wind speed, RPM, and power production measured at each wind turbine.

### Study design

We selected 16 wind turbines for the study at Blue Creek with two constraints: 1) landowner approval was received and 2) each turbine was > 250 m from the nearest wind turbine. Landowners were compensated for crop loss in the 90-m-radius circle (2.5 ha) (S1 Fig). Within the radius, the vegetation was mowed approximately every two weeks. The plots were cleared of crops and mowed on the same day every 2-weeks throughout the study period. A 250-m turbine separation distance ensured that all bat fatalities discovered could be clearly associated with the experimental wind turbine and not a neighboring one.

We conducted a pre-study statistical power analysis using data collected from earlier studies. The analysis indicated that for our 2x2 factorial experiment, a randomized block design to control variation in mortality among wind turbines would better detect treatment differences than a completely randomized design. On each night, each treatment (**Table 1**) was randomly assigned to 4 wind turbines. Assignment was rerandomized nightly with the constraint that the treatment assigned to each turbine was balanced (four nights of each treatment) every 16 nights. At the beginning of the study the wind energy facility operators were given a schedule of which wind turbines to curtail on which nights.

**Table 1:** Treatment descriptions.

Treatment	Deterrent status	Turbine Rotation when windspeed was between 3.0m/s and 5.0 m/s^*^
Control	Off	Freely rotating
Curtail	Off	<2 RPM
Deterrents	Active	Freely rotating
Curtail & Deterrents	Active	<2 RPM

* All turbines are curtailed below the factory cut-in speed of 3.0 m/s.

Throughout the study NRG Systems monitored the functional status of the deterrents on a nightly basis using a cellular modem connection and noted whether there were known deterrent malfunctions at a wind turbine on any given night. We also examined 10-minute average RPM of each wind turbine in relation to 10-minute average wind speed throughout each night to determine if the curtailment was implemented as assigned. Any deviations were recorded. We analyzed results based on the “effective treatment,” indicating how the turbine and/or deterrent actually operated on a given night regardless of its assigned treatment.

Treatments were implemented nightly from 1 hr prior to sunset to 1 hr post sunrise for 112 nights, from 14 June through 2 Oct 2017. Daily searches for carcasses at each experimental wind turbine began 15 June and ended 3 October 2017. We selected this time period based on fatality data collected at the site in 2012 and 2013 that indicated this time interval represented the period during which the most fatalities occurred. All turbines were searched by human observers every day with randomized searcher rotations. Searchers walked along 5-m-wide transects within a 90-m radius of each experimental wind turbine. We selected relatively large plot sizes to address two potential sources of detection bias: 1) undercounting fatalities that fall farther from the wind turbine when struck near the blade tip, which could lead to an overestimate of the effectiveness of ultrasonic deterrents, and 2) undercounting fatalities that fall farther from the wind turbine when struck on windier nights, which could lead to an overestimate of the effectiveness of the curtailment treatment [19].

### Data description

For each study night we summarized the average wind speed and the proportion of the night (from data measured in 10-minute intervals) during which RPM was more than 2 for each wind turbine. We chose a cutoff value of 2 RPM to indicate when a wind turbine was curtailed because it represented a natural break in the data (fully operational wind turbines never fell below 3 RPM). We used 10-minute RPM vs. 10-minute wind speed graphs produced for each wind turbine to ascertain whether curtailment was appropriately applied, recoding treatment to effective treatment when disagreements occurred. We did the same for ultrasonic deterrents. We recorded a count of carcasses in each species group at each wind turbine on each night.

### Data analyses

#### Testing assumption that bat mortality is independent of average wind speed.

Because our experiment involved acoustic deterrents, we could not directly monitor bat activity using acoustic signals. We tested our assumption that bat mortality, not activity, was independent of wind speed using a chi-squared test to determine if bat mortality occurring at non-curtailed wind turbines was proportional to wind speed availability. To achieve this, we quantified number of nights with an average wind speed within one of eight wind speed bins (0.0–3.0 m/s, 3.1–4.0 m/s, 4.1–5.0 m/s, 5.1–6.0 m/s, 6.1–7.0 m/s, 7.1–8.0 m/s, 8.1–9.0 m/s, 9.1–12.0 m/s) and compared the proportion of mortality events in each bin to the proportion of nights with average wind speed in each bin. We restricted analysis to non-curtailed wind turbines because we expected mortality to be disproportionate at lower wind speeds for curtailed wind turbines. We included turbines with active ultrasonic deterrents because we expected a constant proportion reduction in mortality due to ultrasonic deterrents. We combined data into unequal bins to ensure expected cell values ≥5.

#### Testing treatment effects.

To test hypotheses regarding the effects of treatments and wind turbine rotation on bat mortality, we fit generalized linear mixed models to three species groups. In our dataset, eastern red bats were the only high-frequency echolocating species, making it convenient for us to consider bat mortality for a high-frequency species group (containing only eastern red bats), a low-frequency species group that contained all three low-frequency echolocating species (hoary bats, silver-haired bats, and big brown bats), and a third group that considered both high- and low-frequency echolocating species together in an ‘all bats’ group.

For each species group we considered five models: 1) the null model with no covariates, 2) the factorial experiment with indicators of effective curtailment and deterrent and their interaction, 3) a model with a continuous covariate (log(PropSpin)) measuring the proportion of the night during which the wind turbine was rotating > 2 RPM (log-transformed), 4) a model that included a constant effect of deterrent on the relationship of mortality to log(propSpin), and 5) a “comparison of regression lines” model that allowed the effect of ultrasonic deterrents to differ as the proportion of the night during which the wind turbine was rotating > 2 RPM increased. No adjustments for detection probabilities were necessary because all carcasses at any given wind turbine (block) are assumed to be equally detectable. Blocking on wind turbine accounts for variation among wind turbines in mean mortality as well as detectability.

In all our models, the fatality count, Y, at each turbine on each night was assumed to be distributed as a negative binomial, with parameters μ and κ and expected value η:


Y ~ NB(μ,κ), E(Y) = η

Our factorial experiment model took the form of:


$$log\left(\eta \right)\;\sim \;C\;+\;D\;+\;C*D\;+\;I_{turbine}$$

where C is an indicator of curtailment, D is an indicator of active ultrasonic deterrents, and *I*_*turbine*_ is a normally distributed random effect of turbine. We estimated the ratio of mortality for each treatment combination to the mortality observed at the control wind turbines. This model is consistent with many in the mortality-minimization literature and fails to address study-specific conditions of how often curtailed wind turbines operated differently from fully operational wind turbines.

Our model of mortality directly related to the proportion of a night that a wind turbine was rotating took the form of:


$$log\left(\eta \right)\;\sim \;log\left(PropSpin\right)\;+\;I_{turbine}$$

where log(PropSpin) is the proportion of the night (log-transformed) during which the wind turbine was spinning at > 2 RPM. We log-transformed the proportion to maintain the linear relationship between mortality and the proportion of time spinning, and we added a small constant (0.007, that represented half the minimum increment) to accommodate zero fatality counts.

Our model of ultrasonic deterrents having a constant effect on mortality as it directly related to the proportion of a night a wind turbine was rotating took the form of:


$$log\left(\eta \right)\;\sim \;log\left(PropSpin\right)\;+\;D\;+\;I_{turbine}$$

where all variables are defined as above.

Our final model considered the possibility that the effect of active ultrasonic deterrents might differ as the proportion of the night during which the wind turbine was rotating >2 RPM increased. This was expressed in our model as the potential interaction of log(PropSpin) with the indicator of deterrent treatment:


$$log\left(\eta \right)\;\sim \;log\left(PropSpin\right)\;+\;D\;+\;log\left(PropSpin\right)\;*\;D\;+\;I_{turbine}$$

where all variables are defined as above.

We used Akaike information criterion corrected for small sample size (AICc, [20]) to compare these five models and determine which provided the best explanatory power for each species group. Before interpreting AICc results, we used a function in the DHARMa package [21] to simulate residuals and checked models for uniformity, outliers, dispersion, consistency of quantiles, and zero-inflation. We conducted all analyses in program R version 4.2.2 [22].

## Results

### Final data set

Each night, 8 of 16 study turbines were scheduled to have ultrasonic deterrents turn on, for a total of 896 turbine-deterrent nights (8 turbines * 112 nights). We experienced no issues with deterrent functionality on 883 (99%) of the turbine-deterrent nights. Of the 13 nights on which we experienced issues, the entire system (all 6 deterrent units) failed on only four nights). Three of these occurred at one wind turbine that experienced an issue with the circuit breaker. The fourth night the cellular connectivity prevented communications to the deterrent system when trying to reconfigure the schedule at a wind turbine. On nine turbine-deterrent nights, only one of the six units failed; seven of these occurred at one wind turbine. On two turbine-deterrent nights the deterrent units were on when they were scheduled to be off.

Our experiment ran for 1,792 turbine-nights (16 turbines * 112 nights) but our final dataset comprised 1,727 turbine-nights. We could not conduct 62 (3.5%) planned searches due to weather, pesticide applications, or other uncontrollable factors and we excluded 3 turbine-nights because wind turbines were down for repairs and not functioning, or RPM data were not recorded. On 20 of the 1,727 turbine-nights (1%,) the wrong treatment was implemented (Table 2). Such misalignments can occur when wind turbines assigned to curtail do not receive the signal to feather the blades below the curtailment wind speed or wind turbines assigned to not curtail may erroneously receive a signal to curtail. Plots of wind turbine rotor RPM vs. wind speed revealed whether the assigned curtailment treatment was correctly implemented on any given night.

**Table 2:** Number of nights after which searches were conducted where assigned treatment (columns) was the same as (diagonal) or different from (off-diagonal) the effective treatment (rows). The 65 missing values account for the imbalance in treatment assignments.

	Control	Curtail	Deterrent	Curtail & Deterrent
**Control**	436	3	8	0
**Curtail**	1	423	0	2
**Deterrent**	0	0	422	1
**Curtail & Deterrent**	0	2	3	426

During the 112-night study period, we found 234 bats at experimental wind turbines, 154 of which we considered to be “fresh,” i.e., thought to have been killed the previous night and therefore associated with the treatment conditions of the previous night. Upon finding a carcass, survey personnel determined the species and used multiple cues including fluid in the eyes, insect load, odor, and wing pliability to classify whether a carcass was fresh or not. These 154 fresh carcasses comprised five species. Eastern red bat (*Lasiurus borealis*) accounted for the majority of carcasses (n = 86, 56%) followed by hoary bats (n = 30, 20%), silver-haired bats (*Lasionycteris noctivagans*) (n = 20, 13%), and big brown bats (*Eptesicus fuscus*) (n = 17, 11%). There was a single evening bat (*Nycticeius humeralis*). The evening bat was included in the analysis of ‘all bats,’ but not included in the analysis of high-frequency calling bats, as all other carcasses in that group were from a single species, eastern red bat.

### Proportion of night wind turbines rotated >2 RPM

**Fig 1:**
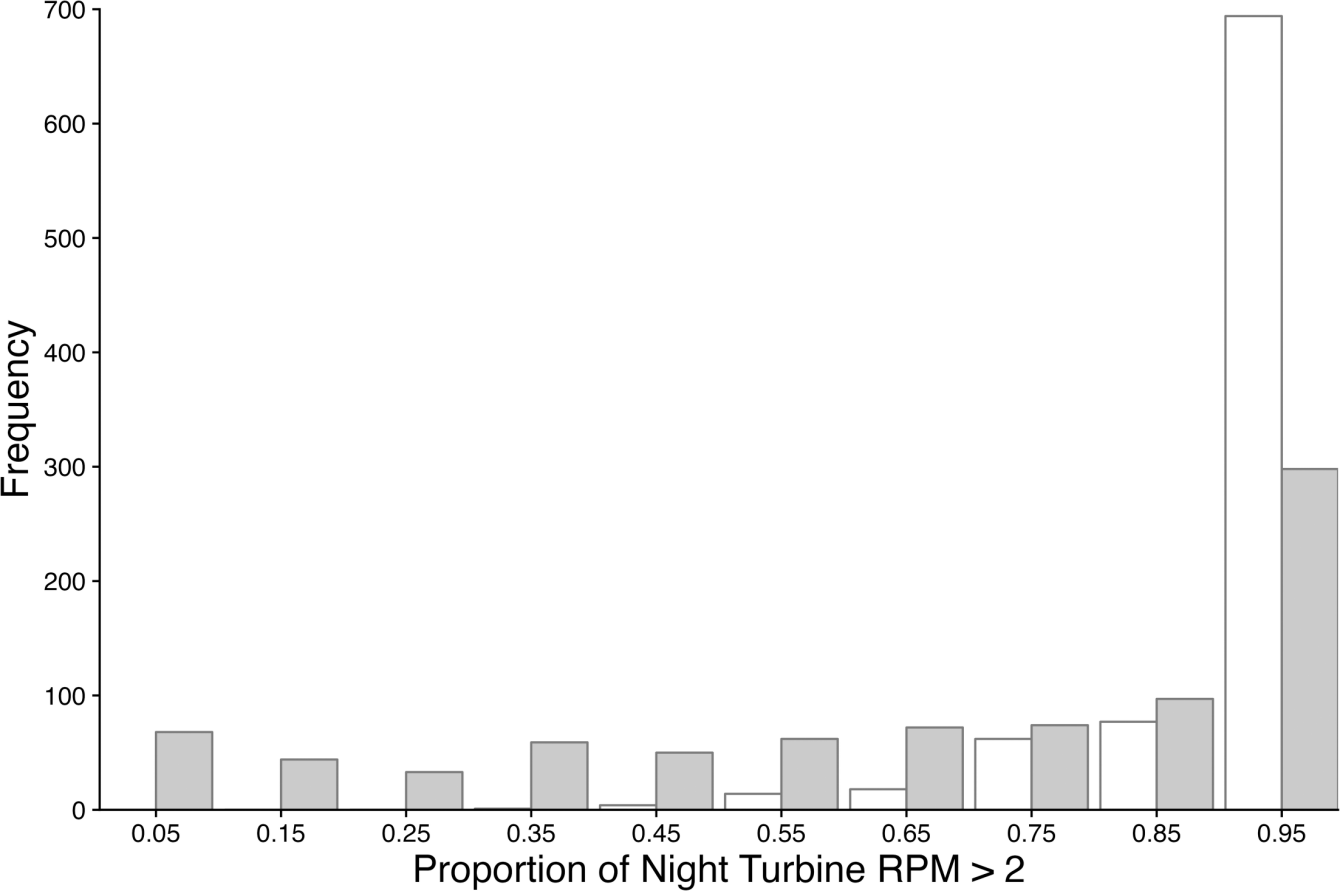
Number of nights with the proportion of the night wind turbines rotated for non-curtailed (open) and curtailed (gray) wind turbines.

The curtailment treatment was only realized when wind speed was between 3.0 and 5.0 m/s. The operation of wind turbines assigned curtailment treatment differed from that of fully operational turbines only 22.0% of the time as measured in 10-minute increments. Wind turbines with curtailment treatment rotated all night 23% of the time, while wind turbines without curtailment treatment rotated all night 65% of the time and never rotated less than 38% of the night (Fig 1).

### Testing assumption that bat mortality is independent of average wind speed

**Fig 2:**
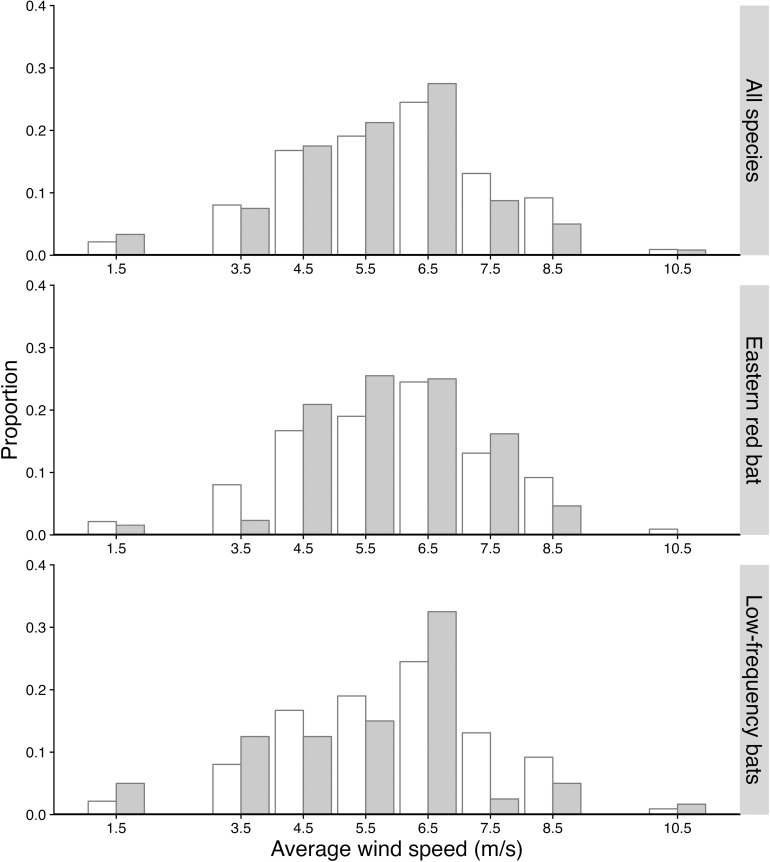
Histogram of the proportion of nights (open) and proportion of fatalities (gray) in each average wind speed bin (0.0–3.0 m/s, 3.1–4.0 m/s, 4.1–5.0 m/s, 5.1–6.0 m/s, 6.1–7.0 m/s, 7.1–8.0 m/s, 8.1–9.0 m/s, 9.1–12.0 m/s).

We found no strong evidence that mortality of all bats combined depended on wind speed ( $\chi_{7}^{2}$ = 4.98, *p* = 0.654) (**Fig 2**). In other words, bats were active and at risk during essentially all wind speeds experienced during the course of this experiment. Sample size limitations required that we bin data when average wind speed was < 3 m/s and when average wind speed was > 9 m/s. It should be noted, however, that on only 6.4% and 2.8% of the nights was the average wind speed <3 m/s or > 9 m/s, respectively. When considered separately, there was again little evidence that eastern red bat mortality depended on wind speed ( $\chi_{7}^{2}$  = 10.87, *p* = 0.133); however, there was some evidence that low-frequency-calling bat mortality depended on wind speed ( $\chi_{7}^{2}$  = 16.27, *p* = 0.026), with disproportionately higher mortality at the lowest average wind speeds (Fig 2).

### Testing treatment effects

All five models passed all lack-of-fit tests. For all three species groups the log of the proportion of the night that RPM > 2 (log(PropSpin)) was included as a covariate for all best models and always had a positive effect on bat mortality (Table 3). The ultrasonic deterrent covariate was also in the best model for the all-species group and eastern red bats and was less than 1  Δ  AICc from the top model for the low-frequency species group (Table 3). For none of the groups was the factorial treatment model within < 4  Δ  AICc units.

**Table 3:** AIC model comparisons within each species group. Group = species group analyzed: eastern red bats (a high-frequency-calling species), low-frequency-calling species (hoary bats, silver-haired bats, and big brown bats), and all species combined (this included a single evening bat as well as the eastern red bats, hoary bats, silver-haired bats, and big brown bats). In the Model column, D = active ultrasonic deterrent, C = curtailment, and PropSpin = proportion of the night during which the wind turbine was spinning at > 2 RPM.

Group	Model	AIC	loglik	Deviance	k	Δ AICc
All	D + log(PropSpin)	1038.71	-514.36	1028.71	5	0.00
All	log(PropSpin)	1038.96	-515.48	1030.96	4	0.25
All	D x log(PropSpin)	1040.09	-514.05	1028.09	6	1.38
All	C x D	1062.86	-525.43	1050.86	6	24.15
All	null	1066.20	-530.10	1060.20	3	27.49
Eastern red bat	D + log(PropSpin)	659.21	-324.60	649.21	5	0.00
Eastern red bat	D x log(PropSpin)	661.02	-324.51	649.02	6	1.81
Eastern red bat	log(PropSpin)	666.12	-329.06	658.12	4	6.91
Eastern red bat	C x D	685.71	-336.86	673.71	6	26.50
Eastern red bat	null	690.22	-342.11	684.22	3	31.01
Low freq spp	log(PropSpin)	570.19	-281.10	562.19	4	0.00
Low freq spp	D + log(PropSpin)	570.87	-280.44	560.87	5	0.68
Low freq spp	D x log(PropSpin)	572.86	-280.43	560.86	6	2.67
Low freq spp	C x D	574.66	-281.33	562.66	6	4.47
Low freq spp	null	576.00	-285.00	570.00	3	5.81

**Fig 3:**
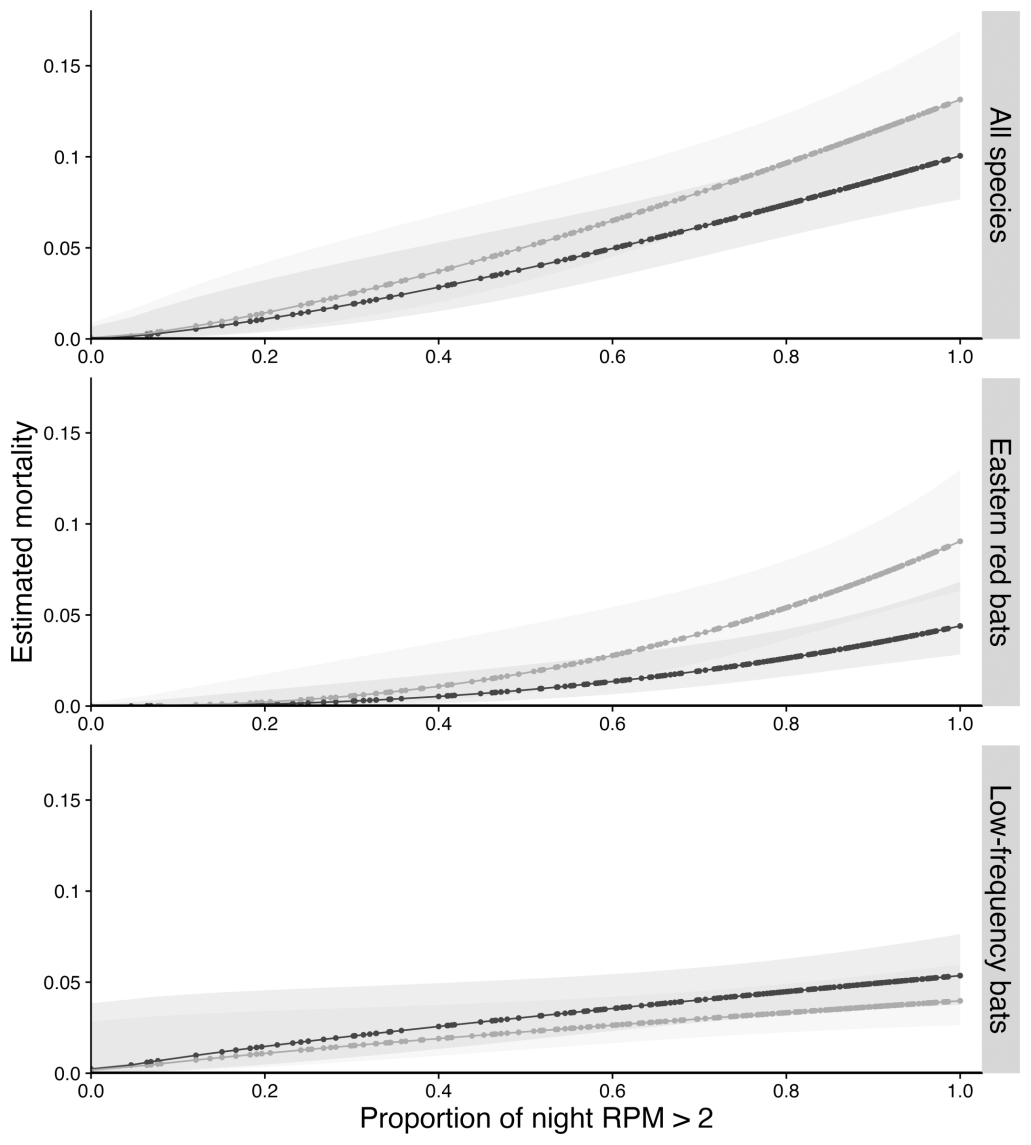
Relationship between estimated nightly mortality at each turbine and the proportion of the night that turbines rotated for turbines with active (gray dots) and inactive (black dots) ultrasonic acoustic deterrents. Gray shading represents 95% confidence intervals. Low-frequency group included hoary bats, silver-haired bats, and big brown bats.

Our models for all 3 species groups indicated that the mortality rate increased as the proportion of the night that wind turbines were spinning increased (Table 4, Fig 3). There was some indication that ultrasonic deterrents had a damping effect on mortality for the all-bats group and the low-frequency-calling species. Counter to expectation, ultrasonic deterrents had an amplifying effect on mortality of eastern red bats. When turbines were spinning all night, eastern red bat mortality was estimated to be 2.08 times as high (95% CI: 1.26, 3.45) when ultrasonic deterrents were active as when they were not (Table 4, Fig 3).

**Table 4:** Coefficient estimates from factorial model and additive deterrent and proportion spinning model for each species group. Note: In this model, the intercept represents a night when turbines are spinning 100% of the time: log(proportion of night spinning) = log(1) = 0. Also note, the D + log(PropSpin) was the best model for the all-bat group and eastern red bats but was second best for the low frequency calling species group. Low-frequency group included hoary bats, silver-haired bats, and big brown bats.

		All bats		Eastern red bat		Low-frequency bats	
Model	Parameter	Est	StdErr	Est	StdErr	Est	StdErr
Treatment Only	Intercept	-2.3	0.17	-3.26	0.27	-2.81	0.21
	Curtailment	-0.62	0.28	-0.45	0.41	-0.76	0.36
	Deterrent	0.16	0.23	0.64	0.32	-0.41	0.33
	CurtxDet	-0.19	0.25	0.37	0.34	-0.86	0.37
D + log(PropSpin)	Intercept	-2.31	0.14	-3.15	0.22	-2.94	0.18
	Deterrent	0.27	0.18	0.72	0.25	-0.3	0.26
	lpropSpin	1.39	0.37	2.35	0.69	0.8	0.39

The best model for the all-species group was one in which mortality increased as the proportion of time a turbine was spinning increased, with deterrent potentially causing a constant proportional drop in mortality (Table 3). The lack of strong effect of ultrasonic deterrents was evident in that the model without the deterrent effect was also a highly competing model ( Δ  AICc = 0.25). For the all-species group, the factorial treatment model was  > 25 delta AICc units from the best and < 5  Δ  AICc units from the null, indicating little strength for this model.

**Fig 4:**
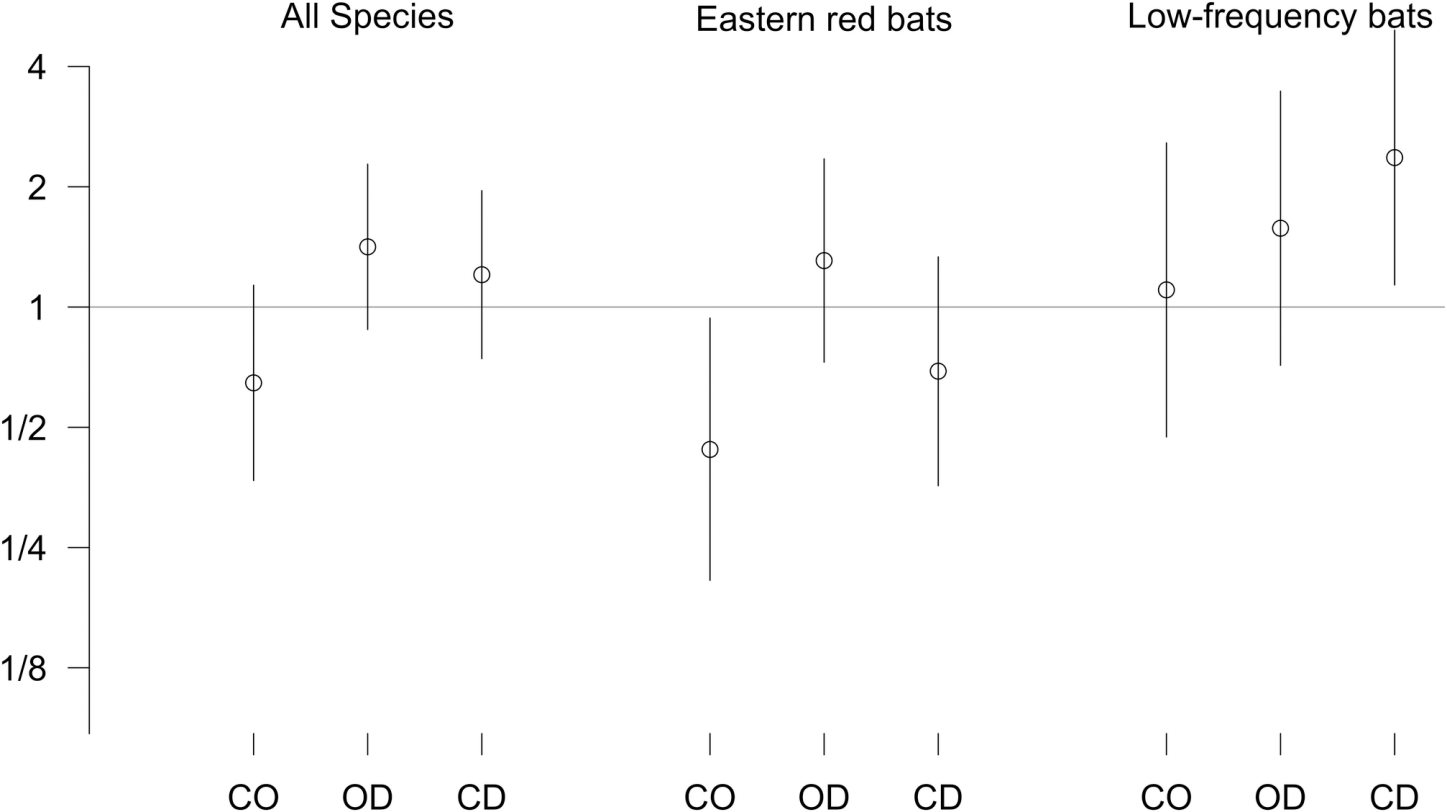
Effect (ratio of estimated mortality for control:treatment) and 95% confidence intervals of each treatment combination on three bat groups between 14 June and 3 October 2017 at the Blue Creek Energy Project. Y-axis is on log(2) scale, and ranges from treatment mortality 1/8 that of control to 4 times that of control. All = all bat species, LABO = eastern red bat, low-freq = big brown bat, hoary bat, silver-haired bat, combined. CO = curtailment only (5.0 m/s), DO = deterrent only, CD = curtailment (5.0 m/s) and deterrent.

For comparison to many earlier studies, we present results from the 2-way factorial model with treatment combination as the only predictor. Under this model, curtailment was estimated to reduce mortality in all three species groups, although its effect was equivocal for eastern red bats (Fig 4). For low-frequency-calling bats, while ultrasonic deterrents alone appeared to reduce mortality, they contributed no additional reduction when combined with curtailment. The effect of ultrasonic deterrents on eastern red bats was contrary to our expectations but consistent with the previous model results. Mortality of eastern red bat mortality was 1.9 times as high (95% CI: 1.01, 3.55) when ultrasonic deterrents were active as when they were not (Table 4, Fig 4).

## Discussion

In this study, we found that the effectiveness of ultrasonic deterrents and/or curtailment below 5 m/s for reducing mortality differed between the two species groups we examined: eastern red bats, a high-frequency calling bat, and the low-frequency calling species group comprised of hoary bats, silver-haired bats, and big brown bats. For both groups, mortality was found to be best explained by the proportion of time the turbines were spinning rather than a simple indicator of whether the turbine was assigned to the curtailment treatment, although the strength of this relationship was much higher for eastern red bats than for the low-frequency-calling group.

We found little evidence that mortality of eastern red bats occurred disproportionately at lower wind speeds ($\chi_{7}^{2}$ *p-value* > 0.1), i.e., eastern red bats were active and killed at essentially all wind speeds. If our finding that mortality is proportional to wind speed availability holds across other wind energy facilities and regions, it implies that raising the cut-in speed will only ever be as effective as the proportion of time the site experiences wind speeds below the designated cut-in speed. In this study, curtailed turbines were spinning 30% less often than control turbines were, and mortality at curtailed turbines was estimated in the treatment-only model to reduce eastern red bat mortality by 36% (95%CI: 71% decrease to 42% increase) (Table 4). However, for eastern red bats, the model directly relating mortality to the proportion of time turbines were spinning, had much more support than the treatment-only model ( Δ  AICc > 25). If mortality is better explained by the proportion of time that turbines are spinning (closely related to proportion of time that wind speed is greater than the cut-in speed), then we expect lower mortality reduction due to curtailment at wind facilities with higher average wind speeds, or equivalently, those at which turbines are rotating a higher proportion of time.

The pattern of independence of mortality and wind speed observed for eastern red bats did not hold for low-frequency-calling bats ($\chi_{7}^{2}$ *p* = 0.026); instead, mortality was disproportionately higher at the lowest wind speeds. In the treatment-only model, curtailment alone was estimated to reduce low-frequency-calling bat mortality by 53% (95% CI: 5%, 77%) (Table 4), considerably higher than the reduction in proportion of time curtailed turbines were spinning relative to control, but consistent with the lack of independence of wind speed and mortality for this group. Although for the low-frequency-calling bats, the model directly relating mortality to the proportion of time turbines were spinning had more support than the treatment-only model, it was not strong (Table 3). This indicates that for bat species that fly disproportionately more often at lower wind speeds, curtailment at lower wind speeds may be more effective than for other species.

As would be expected, when all bat species were combined into one group (n = 87 high-frequency-calling bats (n = 86 eastern red bats + 1 evening bat), n = 67 low-frequency-calling bats), the estimated mortality reduction due to curtailment was intermediate between the two groups (46% (95% CI: 7%, 69%); Table 4). The model directly relating mortality to the proportion of time turbines were spinning, however, had much more support than the treatment-only model. These results underscore the importance of analyzing effects of minimization measures on individual species or behavioral groups and resisting the temptation to assume all bats will respond similarly.

The eastern red bat response to ultrasonic deterrents was opposite of what we predicted, with mortality estimated to be 105% (95%CI: 26%, 235%) higher when ultrasonic deterrents were active, and turbines were spinning all night. The estimate from the treatment-only model was similar, 90% higher (95%CI: 1%, 255%), but the model had far less support (Table 3). The response to ultrasonic deterrents of the low-frequency-calling bats was equivocal, with estimated effect between 55% reduction and 23% increase in mortality. In addition, contrary to results at other sites with different species composition, and/or deterrent configurations [3,10,18], we found that the effect of mortality reduction due to ultrasonic deterrents was not additive relative to curtailment for low-frequency-calling bats, i.e., mortality at curtailed wind turbines with active ultrasonic deterrents was essentially the same as mortality at curtailed wind turbines without active ultrasonic deterrents. Our result suggests that ultrasonic deterrents may not be more effective than curtailment for either species group and especially eastern red bats.

Prior to validation studies at wind energy facilities, Spanjer [23], Szewczak and Arnett [24], among others conducted laboratory and field studies on the responses of bat activity to ultrasonic deterrent in natural settings. Working at ponds, Szewczak and Arnett [unpublished] demonstrated that ultrasonic deterrents elicited a strong negative response on overall bat activity rates within a distance of ~ 20 m. Gilmour et al. [24] also observed a reduction in activity of free-flying bats. Zeale et al. [25] and Aldemir Bektas et al. [26] found evidence that ultrasonic deterrents appear to temporarily deter bats from historic buildings and bridges. Why ultrasonic deterrents perform well outside of wind energy facilities relative to the mixed results produced at wind energy facilities remains unknown, but explanations may include the challenges of covering such a large structure given the constraints of ultrasound propagation and/or differences in behavioral expressions of bats when interacting with wind turbines versus more natural habitats. Further, ultrasound may be more effective initially as a way to startle bats, after which bats become habituated to exposure [27]. Knowing where on the blade collisions occurred would help address whether ultrasonic deterrents, rather than preventing bats from entering the rotor-swept area altogether, are instead simply causing bats to fly farther from the nacelle but still be at risk within the rotor swept area.

Eastern red bats were the only high-frequency echolocating species in our study. Because ultrasound attenuates more rapidly as frequency increases, eastern red bats may move through more of the rotor-swept area prior to perceiving ultrasound which could disrupt their ability to effectively echolocate and deter their approach. Alternatively, eastern red bats may simply behave differently at wind turbines than the three low-frequency-calling species in our study, perhaps decreasing reliance on echolocation and increasing reliance on visual cues. Our findings suggest the need for caution when deploying ultrasonic deterrents at sites with species that deterrent effects have not been studied.

This counterintuitive result raises additional questions about the character of ultrasonic deterrent pulse rates and repertoires. Current ultrasonic deterrents emit constant-frequency noise across several frequency ranges and seek to disrupt a bat’s ability to echolocate; however, many bat species rely on vision as much as echolocation, and outside of certain contexts, such as those that might be more likely at ponds or roosting sites (e.g., foraging, fine scale flight maneuvers), bats may prioritize visual cues. If visual cues are prioritized during some wind turbine interactions, we might explore ultrasonic deterrents that use novel pulse rates to effectively startle and deter bats rather than to jam their echolocation abilities. Further, we must consider that the perceived value of whatever is attracting bats to wind turbines may outweigh the costs of having to navigate through a noisy environment. If we can identify wind turbine attractants and reduce their attractiveness to bats, we may find that ultrasonic deterrents will then be more effective.

Overall, we found that ultrasonic deterrents did not significantly improve mortality reduction beyond control conditions and may have alternatively caused an increase in eastern red bat mortality. It remains unclear why eastern red bat mortality was higher in our study when ultrasonic deterrents were active, but it underscores the dangers of assuming similarity of species’ responses and inferring applicability of results to all species.

## Supporting information

S1 Fig Avangrid Renewables’ Blue Creek Wind Energy Facility in Van Wert and Paulding counties, Ohio. Basemap provided by ESRI licensed under the Esri Master License Agreement. https://server.arcgisonline.com/ArcGIS/rest/services/World_Topo_Map/MapServer
(DOCX)
