# Supplementary figures and images for: Ultrasonic deterrents provide no additional benefit over curtailment in reducing bat fatalities at an Ohio wind energy facility
Source: PLoS One. 2025 May 8;20(5):e0318451. doi: 10.1371/journal.pone.0318451 (PMC12061157; doi:10.1371/journal.pone.0318451)

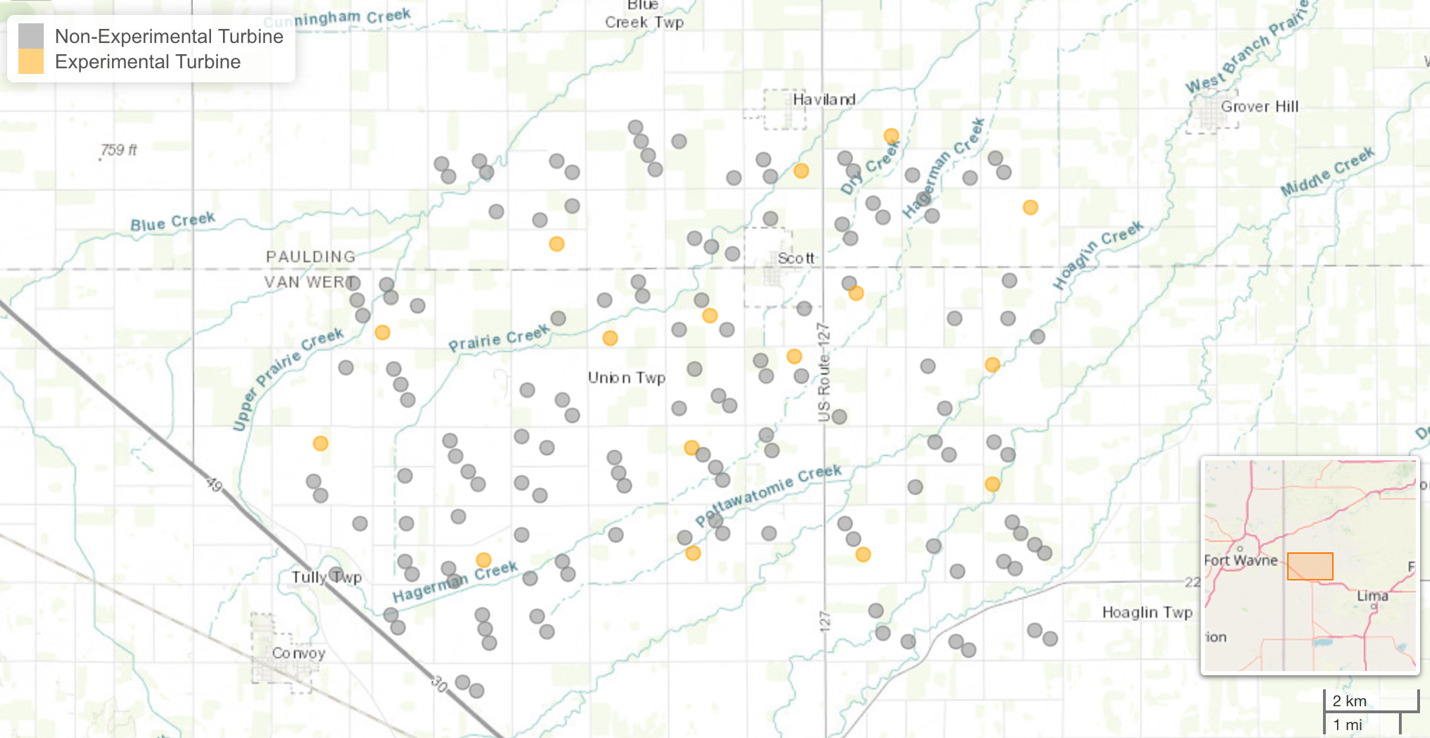

Supplement: S1 Fig — Basemap provided by ESRI licensed under the Esri Master License Agreement. https://server.arcgisonline.com/ArcGIS/rest/services/World_Topo_Map/MapServer (DOCX) [file pone.0318451.s001.docx]
